# Maintenance of Genetic Diversity in an Introduced Island Population of Guanacos after Seven Decades and Two Severe Demographic Bottlenecks: Implications for Camelid Conservation

**DOI:** 10.1371/journal.pone.0091714

**Published:** 2014-03-24

**Authors:** Benito A. González, Pablo Orozco-terWengel, Rainer von Borries, Warren E. Johnson, William L. Franklin, Juan C. Marín

**Affiliations:** 1 Laboratorio de Ecología de Vida Silvestre, Facultad de Ciencias Forestales y de la Conservación de la Naturaleza, Universidad de Chile, Santiago, Chile; 2 School of Bioscience, Cardiff University, Cardiff, Glamorgan, Wales, United Kingdom; 3 Facultad de Medicina, Universidad de Chile, Santiago, Chile; 4 Smithsonian Conservation Biology Institute, Smithsonian Institution, Front Royal, Virginia, United States of America; 5 Department of Natural Resource Ecology and Management, Iowa State University, Ames, Iowa, United States of America; 6 Laboratorio de Genómica y Biodiversidad, Facultad de Ciencias, Universidad del Bío-Bío, Chillán, Chile; Univerity of Puerto Rico at Mayaguez, United States of America

## Abstract

Fifteen guanacos were introduced to Staats Island in the Falklands/Malvinas archipelago from Patagonia in the 1930s. Twenty five years later, the population was culled from 300 to 10–20 individuals, but quickly rebounded to a population of almost 400 animals that today retain the genetic signature of the founding event and later bottleneck. The goals of this study were to (i) make a genetic assessment of this island population through comparisons with mainland populations and simulations, and (ii) assess the likely source-population of the introduced guanacos. Genetic variation was estimated from 513 bp of mitochondrial DNA sequence and 15 microsatellite loci among 154 guanacos collected from eight localities, including the adjacent mainland and the islands of Tierra del Fuego and Staats Island. Of the 23 haplotypes observed among our samples, the Staats Island population only contained three haplotypes, all of which were shared with the coastal Monte Leon population in southern Patagonia. Mitochondrial DNA and microsatellite variations on Staats Island were comparable to most mainland populations and greater than those observed on Tierra del Fuego. Patterns of genetic structure suggest that the Staats Island guanaco population was founded with animals from southern Patagonia (as opposed to northern Patagonia or Tierra del Fuego), but that effective reductions in population size lasted only a few generations and that surviving animals were a random sample of the pre-bottleneck genetic variation.

## Introduction

Genetic analysis is a well-used tool for the conservation and management of animal populations and can help in determining genetic patterns, differentiating populations, resolving taxonomic uncertainties, and addressing evolutionary questions [1]–[5]. Small populations of endangered species have particularly benefited from genetic surveys and have contributed to the design of management strategies for breeding and reintroductions into the wild [6]–[8]. Genetic studies have also had an important management role in describing the effects of demographic changes (e.g. founder effects, bottlenecks) on the genetic variation of populations after capture, translocation, and release of wild individuals [9]–[15]. However, few studies have genetically surveyed wild or endangered species with well-documented population dynamics in order to test hypotheses about the impact of bottlenecks on genetic variation, and subsequent population persistence [16].

The guanaco (*Lama guanicoe*) is a native ungulate of South America. It is distributed widely from Perú (8° S) in the north, through Bolivia, Paraguay and Argentina until the southernmost part of Chile (55°S) [17], [18]. Large guanaco populations occur from sea level to nearly 5000 m elevation [19], inhabiting deserts, some arid portions of the Andean mountains, and the shrublands and steppe plains of Patagonia [17], [20]. A large population is also found on the large island of Tierra del Fuego [17], [21]–[23], possibly arriving there at the time when sea levels were lower at the end of the Pleistocene 10,000-11,000 years ago and land bridges spanned over the Magellan Strait [24], [25]. Besides the Tierra del Fuego population, the only other natural island population occurs on Navarino Island [26], but how guanacos spread from the continent to this island is still unclear [27].

The guanaco has successfully colonized new and marginal areas because of its capacity for adapting to an array of arid environments characterized by low annual rainfall, little-to-no winter snow-cover, low primary productivity, and high seasonality [17]. The species has been re-introduced to regions where natural populations were highly reduced or extirpated either by anthropogenic causes or natural events, such as, volcano eruptions and fires [28]–[32]. Various characteristics of guanaco biology (e.g. longevity, fertility, reproductive strategies, flexible social groupings, generalist-feeding habits, and a mating-birthing season encompassing the best weather and vegetation-growing season) have contributed to its ability to survive in small isolated populations [17], [20], [29], [33].

However, the genetic impacts of these population dynamics have not been studied in this species. Recent genetic studies using mitochondrial DNA indicate that on a broad geographic scale, the guanaco is a monophyletic species with genetic lineages that support the previous classification of two subspecies: *L. g. cacsilensis* and *L. g. guanicoe* [34]–[36]. On a finer scale, microsatellite-based comparisons among populations on the South American mainland and Tierra del Fuego show that the guanaco is a diverse species with low to moderate population structure [25], [36], [37].

The guanaco population on Staats Island, in the Falkland Island archipelago (also known as Islas Malvinas) in the South Atlantic Ocean, offers a natural and on-going laboratory for testing the effects of a founder event on a guanaco population. This unique population has remained small and completely isolated from other populations for over 70 years. Together with several other Patagonian species, guanacos were introduced in the late-1930s by John Hamilton in an attempt to diversify the local economy [38]. Young animals were plausibly captured near Rio Gallegos (Argentina), or Pali-Aike (Chile) on the South American mainland, and then shipped to Sedge Island (11 animals) and Staats Island (15 animals) in the Falkland/Malvinas archipelago [38]. Only the guanacos on Staats Island survived despite several attempts 25 years later to eradicate them to reduce overgrazing and convert the island to sheep husbandry. This severe culling in the late-1950s drastically reduced the population from 300 animals to its second bottleneck and smallest size of 10 to 20 individuals in the early 1960s [38], [39]. The population was then permitted to increase, with only sporadic and low levels of poaching and culling [38], [39]. In 2004 the population numbered approximately 400 guanacos [40]. To best understand and interpret the relative genetic status and health of the island population, we compared it with mainland-guanaco populations of southern Patagonia.

Here we present the first genetic assessment of a small-island population of guanacos. We hypothesize that the population is characterized by low levels of genetic diversity as a result of two demographic bottlenecks in its short history as impacted by inbreeding, when each time it was reduced to less than 20 surviving animals. We also establish the phylogenetic relationship between this population and mainland populations in order to determine the most likely source of guanaco-genetic variation on Staats Island. Finally, based on these results we propose alternatives for the management of guanaco populations on the continent and Staats Island itself.

## Materials and Methods

### Ethics Statement

Guanaco samples did not come from any endangered guanaco populations in Chile or Argentina, where the guanaco is classified as “Least Concern” by the Red List, IUCN [41]. Liver samples were opportunistically taken collected from carcasses of adults animals hunted for meat production in Valle Chacabuco authorized by Chilean Government. Blood samples were obtained after chemical immobilization of adult guanacos in Torres del Paine National Park and Tierra del Fuego. The present study did not require the capture or handling of animals on Staats Island, Bosques Petrificados, San Julián, Monte León, Pali-Ayke and 8 animals from Tierra del Fuego, because our samples came from faeces and/or carcasses (muscle and skin). Guanaco samples from natural populations were obtained under permits and supervision of Servicio Agrícola y Ganadero (SAG) in Chile (permits Numbers 447, 263 and 1843) and samples from Staats Island and fecal samples obtained in Argentina were imported to Chile for analysis under CITES authorizations (Numbers 22920 and 22967). Samples taken within Chilean National Parks were authorized by Corporación Nacional Forestal (CONAF, permit number 6/02, 2002). DNA sequences were deposited in GenBank (accessing numbers JX678477 - JX678596). Individual-by-individual microsatellities data are available in the Dryad data repository at doi:10.5061/dryad.06g5v.

### Study area

Staats Island is a small (500 ha) isle on the far western edge of the 750-island Falkland/Malvinas archipelago in the South Atlantic Ocean (51°53’ S latitude and 61°11’ W longitude) 600 km from the South American coast. Six research expeditions were conducted on Staats Island in December (early summer and beginning of guanaco birth season) from 1999 to 2008 and biological samples were collected for genetic analyses in 2004 and 2005. Staats Island is treeless and hilly. The south end is dominated by the Staats Plateau, a tableland surrounded by a coastline of formidable cliffs and monoliths. The island is characterized by large sink holes, cuts and gulches that are caused by connections to the sea along the coastline, especially on Staats Plateau. North of the plateau the balance of the island is dominated by domed peaks (max. 140 m) separated by steep slopes and four valleys of short grass and forb meadows. Dominant plant communities are Oceanic Heath, Grass, Cushion Plants, and Greens (meadows known as vegas and mallines on the mainland) [40], [42].

### Sample collection and DNA extraction

**Figure 1:**
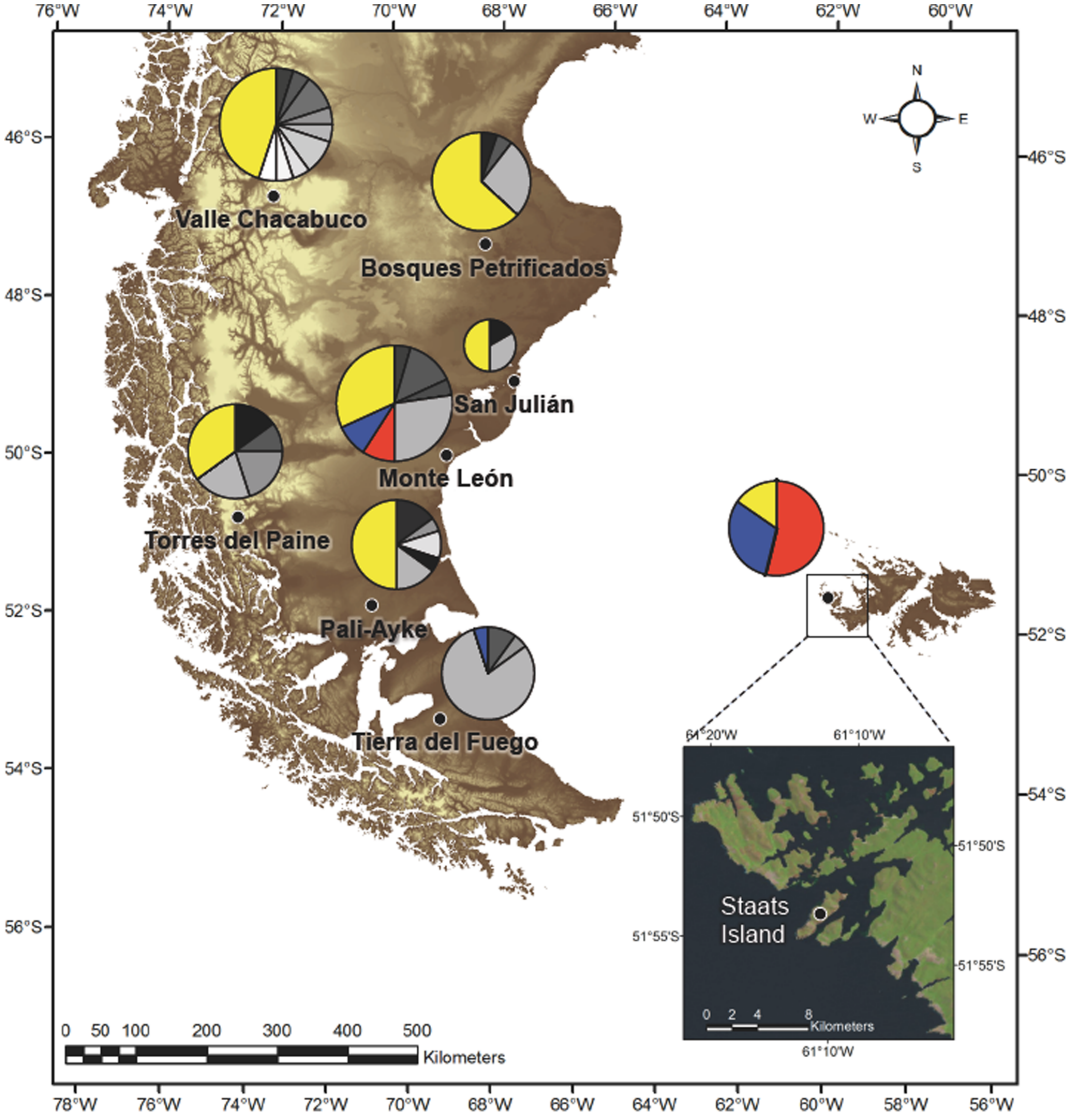
Map of southern South America and the Falkland/Malvinas Islands with sampled localities of *Lama guanicoe* analysed. Each location indicates the relative proportion of each haplotype in yellow, blue and red relative to Staats Island. Mainland haplotypes not found on Staats Island are shown in grey scale. The size of the sphere is proportional to the number of individuals sampled.

For comparison, material suitable for DNA analysis (170 samples) was collected from eight localities throughout southern Patagonia and Staats Island (Figure 1, Table 1). DNA was extracted: i) from muscle or skin samples from 36 dead animals from Staats Island and Tierra del Fuego (Chile), ii) from blood samples of 34 wild-caught adults following chemical immobilization [43], [44] at Torres del Paine National Park and Tierra del Fuego (Chile), and iii) from 74 fresh fecal samples from different dung piles at Bosques Petrificado National Parck, San Julian and Monte Leon (Argentina) and Pali-Ayke National Park (Chile). DNA was also obtained opportunistically from liver samples of 26 adult males in Valle Chacabuco, Chile. Sample sites and the geographic position of individuals collected at each site are given in Figure 1 and Table 1. All samples were stored at –70°C in the Laboratorio de Genómica y Biodiversidad, Departamento de Ciencias Básicas, Facultad de Ciencias, Universidad del Bio-Bío, Chillán, Chile. We followed guidelines of the American Society of Mammalogists during the collection and handling of animals [45]. Total genomic DNA was extracted from blood using the Wizard Genomic DNA Purification Kit (Promega, Madison, Wisconsin). DNA from liver, skin and muscle samples was purified using proteinase-K digestion and a standard phenol-chloroform protocol [46]. DNA from feces was extracted using the QIAamp DNA Stool Mini Kit (QIAGEN, Valencia, California) in a separate non-genetic-oriented laboratory.

**Table 1:** Summary of the *Lama guanicoe* samples used in the genetic analyses, including localities, abbreviations, geographic positions, type of sample (B =  blood, F =  fecal, M =  muscle, S =  skin, and L  = liver), and total number (N) of samples used from each locality for each genetic marker.

Locality, country	Abbreviation	Geographic positions	Sample type	Samples mtDNA (N = 156)	Samples microsatellites (N = 164)
Bosques Petrificados, Argentina	BP	47° 20‘ S, 67° 30’ W	F	20	20
Valle Chacabuco, Chile	VC	47° 36‘ S, 72° 27’ W	L	20	26
San Julian, Argentina	SJ	49° 22’ S, 67° 40’ W	F	8	8
Monte León, Argentina	ML	50° 16’ S, 68° 51’ W	F	22	26
Torres del Paine, Chile	TP	51° 03’ S, 72° 55’ W	B	20	23
Pali-Aike, Chile	PA	52° 04’ S, 69° 47’ W	F	20	20
Tierra del Fuego, Chile	TF	53° 18' S, 70° 11' W	B, S	20	21
Staats Island, United Kingdom	SI	51° 53’ S, 61° 11’ W	M, S	26	20

### Mitochondrial DNA analysis

A 513 bp long fragment of the 5’ side of the mitochondrial Control Region was amplified from 156 samples using the primers LThr-ARTIO 5′ TCC TTT TTC GGC TTA CAA GAC C 3′, Hloop550G 5′ ATG GAC TGA ATA GCA CCT TAT G 3′, Lloop0007G 5′ GTA CTA AAA GAA AAT ATC ATG TC 3′ and H362 5′ GGT TTC ACG CGG CAT GGT GAT T 3’ and H15998 5′ CCA GCT TCA ATT GAT TTG ACT GCG 3′ [34], [35]. The polymerase chain reactions (PCRs) followed standard protocols by Marín *et al.* [34], [35]. All PCR products were purified with the QIAquick PCR Purification Kit (QIAGEN) and sequenced in a ABI-3100 DNA sequencer (Perkin Elmer Applied Biosystems). PCR products were sequenced in both directions twice to test sequence fidelity. Sequences were aligned with Geneious Aligment implemented in Geneious Pro 5.3.4 (Biomatters Ltd.) and the alignment was checked visually. Within population genetic variation was measured by the number of polymorphic sites (S), haplotype number (k), number of private haplotypes, nucleotide diversity (π), and haplotype diversity (H) using ARLEQUIN 3.5.1.2 [47]. Genetic differentiation among populations was expressed as pairwise fixation indices (*Ф_ST_*) calculated with ARLEQUIN 3.5.1.2 [47] using 10,000 permutations to assess significance with K80+I, and the best fit model was tested with jModeltest.

### Microsatellite DNA analysis

Fifteen autosomal dinucleotide microsatellite loci were analysed: YWLL08, YWLL29, YWLL36, YWLL38, YWLL40, YWLL43, YWLL46 [48], LCA5, LCA19, LCA22, LCA23 [49], LCA65 [50], LCA82 [51] and LGU49, LGU68 [52]. Amplification was carried out in a 10 μL reaction volume containing 50 – 100 ng of template DNA, 1.5 – 2.0 mm MgCl_2_, 0.325 μm of each primer, 0.2 mm dNTP, 1X polymerase chain reaction (PCR) buffer (QIAGEN) and 0.4 U *Taq* polymerase (QIAGEN). All PCR amplifications were performed in a PE9700 (Perkin Elmer Applied Biosystems) thermal cycler with the following cycling conditions: initial denaturation at 95°C for 15 min, followed by 40 cycles of 95°C for 30 s, 52–57°C for 90 s and 72°C for 60 s, and a final extension of 72°C for 30 min [36]. Amplification and genotyping of DNA from samples was repeated 3 times. One primer of each pair was labelled with a fluorescent dye on the 5′-end, and fragments were analysed on an ABI-3100 sequencer (Perkin Elmer Applied Biosystems). Data collection, sizing of bands and analyses were carried out using GeneScan software (Applied Biosystems).

We identified multiple samples that came from the same individual by searching for matching microsatellite genotypes using the Excel Microsatellite Toolkit [53] and eliminated samples from the study if they showed more than 85% overlap. We excluded three loci (LCA19, LCA22 and LCA82) after checking for null alleles (i.e. non-amplifying alleles) using Micro-Checker [54]. Within population allele frequencies, Hardy-Weinberg equilibrium (HWE), linkage disequilibrium, observed heterozygosity (*H_O_*), and expected heterozygosity (*H_E_*) were estimated using FSTAT [55]. FSTAT software was also used to estimate population pairwise *F_ST_* values with 10,000 permutations to assess significance. The difference between the expected heterozygosity in Staats Island and mainland populations were statistically tested using Welch’s t-test to account for sample heteroskedasticity utilizing R software (http://www.R-project.org). The inbreeding coefficient *F_IS_* was calculated using GENETIX version 4.05 [56], and its deviation from zero was assessed with 10,000 permutations across loci.

The program STRUCTURE 2.3.3 [57] was used to determine the number of clusters which best partitions the microsatellite data under different scenarios of population independence and admixture. We ran five independent runs assuming no admixture and independent allele frequencies for values of K from 1 to 8 with 200,000 burn-in steps for the MCMC and 200,000 data collection steps. The value of K best representing the division of the samples was identified with the method of Evanno [58].

Three methods were used to assign individuals to the clusters found by STRUCTURE. The first method is based on the probabilities of admixture inferred for each individual by STRUCTURE (*q*). An individual was considered assigned with high confidence if it presented a *q* ≥ 0.75 for a single cluster. The second method consisted of assigning individuals to STRUCTURE’s clusters using the likelihood-based method of [59] implemented in GENECLASS 2.0 [60]. From this analysis, the proportion of individuals assigned to the cluster that they were originally sampled from was reported.

Lastly, we also determined the continental population from which the Staats Island individuals came from using the trained clustering method in BAPS 5.2 [61]. In this analysis the individuals from Staats Island were allowed to cluster to any of the eight continental populations, or alternatively, if these populations did not reflect their population of origin, they were also allowed to form their own cluster. For that purpose we used a prior distribution of the number of clusters in the dataset between 1 and 10, so that the Staats Island individuals could also form new clusters beyond the eight potential clusters formed by the continental populations.

Due to the known changes in population size experienced by the Staats Island population, we also tested whether there was evidence of population bottlenecks in the microsatellite data. For this purpose we used the program BOTTLENECK [60] that aims to detect the excess of heterozygosity left after a bottleneck. These analyses were performed under the stepwise mutation model (SMM [62]) and the multiple step stepwise mutation model (TPM [63]). Under the TPM model the proportion of single-step mutation events was set at 90% with a mutation size-variance of 12%. Observed and expected heterozygosities were compared using a Wilcoxon sign-rank test as suggested by Piry *et al.* [60]. Complementary to the BOTTLENECK analysis, we also tested for deviations between the observed and expected number of alleles in each locus and their size range using the M-ratio method implemented in the M_P_VAL and Critical_M software [64]. During a bottleneck low frequency alleles become extinct, leaving gaps in the allele range distribution of a microsatellite. The ratio between the observed number of alleles and the allele size range is compared to the one expected under mutation-drift-equilibrium using simulations. If 95% of the simulations present a larger ratio (M_c_) than the observed data it is considered that the analysed population passed through a bottleneck. The simulations are performed under the TPM model parameterised by the proportion of single step mutations (*p_s_*), the size of the multistep mutations (Δ_g_) and the neutral evolution rate (θ = 4N_e_μ). As the M_c_ threshold is sensitive to these parameters and there is no species-specific information on N_e_, we performed simulations under various biologically plausible θ values from 0.01 to 20. To ensure this range of values was relevant, we estimated θ from the expected heterozygosity (Eq. 3.15 from [65]) using a common microsatellite mutation rate (μ) recommended by Garza & Williamson [64]: 5.0×10^−4^ mutants/generation/locus [66].

### Simulation of population variation size on genetic diversity

**Figure 2:**
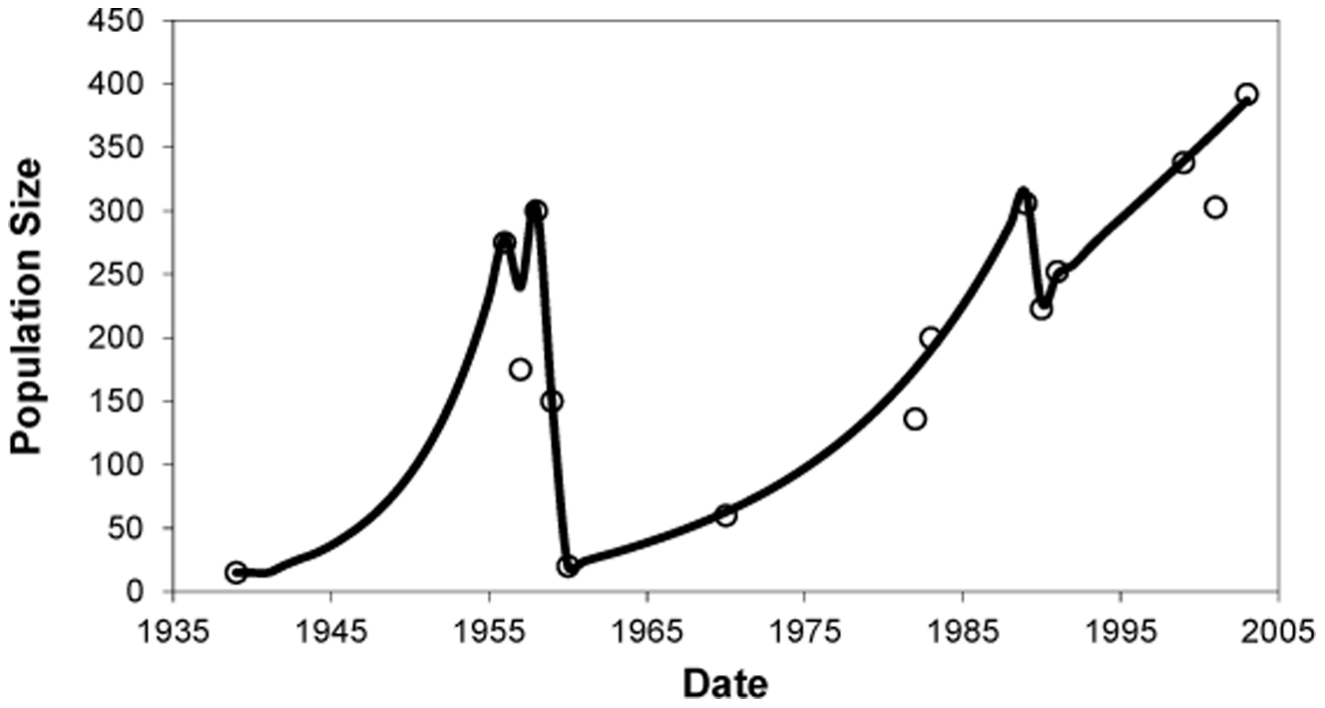
Reconstruction of population size (solid line) of guanacos on Staats Island since introduction based on historical records reported by [38] (circles).

Current genetic diversity of the Staats Island population was compared with those of simulated populations using the software BOTTLESIM [67]. The model was run for 10,000 iterations with non-constant population size, random mating assuming that males have a chance to reproduce at any times of their lives [68]; reproductive maturity at 5-years of age [68], age of senescence ^∼^15 years [21]; 70% generation overlap considering a ratio 4–5/15 (first years of life without reproduction/lifespan). An initial population of 10,000 individuals was constructed with the genetic characteristics of the continental Patagonian population, which was subsequently subjected to a bottleneck that reduced the population size to 15 animals. Temporal variation size of the Staats Island population (Figure 2) was constructed in excel and based on historical records obtained by Franklin and Grigione [38], and was used as a model in post-introduction population size on Staats Island. BOTTLESIM provided means for *H_E_*, and alleles per locus (A) for the initial population and each of the 65 years of simulation.

## Results

### Mitochondrial DNA diversity

Among the 156 samples, there were 23 haplotypes and 15 polymorphic sites (2.9%) from the 514 bp Control-Region fragment (Table 2). A dominant haplotype (H_5) was observed in seven of eight localities, including Staats Island. Haplotype (*h*) and nucleotide diversity (*π*) are detailed in Table 3. As expected, the continental populations presented a higher diversity than the island populations (0.75±0.08, mean and standard deviation of *h*; Table 3). The Staats Island population had a similar diversity (*h* = 0.61) to the Bosques Petrificados population in southern Argentina and was higher than the island population of Tierra del Fuego (*h* = 0.36), which has a large population of more than 80,000 individuals [69], [70]. Of the 23 haplotypes observed among our samples, the Staats Island population only contained three haplotypes, all of which were shared with the Monte Leon population (the only continental population that contained all three haplotypes observed on Staats Island) (Table 2, Figure 1).

**Table 2:** Distribution of the 23 Control Region haplotypes (frequency) observed in 156 guanacos from 8 localities.

Haplotypes						1	1	1	1	2	2	2	2	3	5	Localities								N
			1	4	7	2	7	9	9	1	4	7	8	2	0									
	1	2	5	4	6	5	4	0	1	1	2	0	6	0	8	PB	VC	SJ	ML	TP	PA	TF	SI	
1	C	C	G	A	A	A	T	C	G	C	A	T	C	C	C		1(0.05)							1
2	T	T	.	T	.	.	.	.	A	.	.	.	.	.	.		1(0.05)							1
3	A	T	A	.	.	.	.	.	A	.	.	.	.	.	.		2(0.10)							2
4	A	T	.	.	.	.	.	.	A	.	.	.	.	.	.		1(0.05)				2(0.10)			3
**5**	A	T	.	.	.	.	.	.	.	.	.	.	.	.	.	**12(0.60)**	**9(0.45)**	**3(0.50)**	**7(0.32)**	**7(0.35)**	**10(0.50)**		**4(0.15)**	52
6	A	T	.	T	.	.	.	.	.	.	.	.	.	.	.	5(0,25)	1(0.05)	2(0.30)	6(0.27)	4(0.20)	3(0.15)	16(0.80)		37
7	A	A	.	.	.	.	.	.	.	.	.	.	.	.	.		1(0.05)							1
8	A	T	.	.	.	G	.	.		.	.	.	.	.	.	1(0.05)	1(0.05)		3(0.14)					5
9	A	T	.	.	.	.	.	.	A	.	.	C	.	.	.		2(0.10)							2
10	A	T	.	.	.	.	C	.	.	.	.	.	.	.	.		1(0.05)							1
11	A	.	A	T	G	.	.	.	.	.	.	.	.	.	.	1(0.05)								1
12	A	T	.	.	.	.	.	.	.	.	.	.	G	.	.	1(0.05)								1
**13**	A	.	.	T	.	.	.	T	.	.	.	.	.	.	.				**2(0.09)**				**14(0.54)**	16
14	A	.	.	T	.	.	.	.	.	.	.	.	.	.	.			1(0.20)		4(0.20)	1(0.05)			6
15	A	.	.	T	.	G	.	.	A	.	.	.	.	.	.				1(0.05)					1
**16**	A	T	.	T	.	.	.	.	A	.	.	.	.	.	.				**2(0.09)**			**1(0.05)**	**8(0.31)**	11
17	A	.	.	T	G	.	.	.	.	.	.	.	.	.	.				1(0.05)					1
18	A	.	.	T	.	.	.	T	.	.	.	.	.	T	.					2(0.10)				2
19	A	T	.	.	.	.	.	.	.	T	G	.	.	.	.					3(0.15)				3
20	A	T	.	T	.	G	.	.	.	.	.	.	.	.	.						3(0.15)			3
21	A	T	.	T	.	.	.	T	.	.	.	.	.	.	.						1(0.05)			1
22	A	T	.	T	.	.	.	.	.	.	.	C	.	.	.							2(0.10)		2
23	A	T	.	T	.	.	.	.	.	.	.	.	.	.	G							1(0.05)		1
Total																20	20	6	22	20	20	20	26	154

The vertical numbers indicate the position of polymorphic sites relative to haplotype 1. Bold numbers emphasized the haplotypes presented in Staats Island.

**Table 3:** Genetic diversity indices from mtDNA Control Region sequences and 12 microsatellite loci by localities (defined in Table 1 and Figure 1).

Localities	mtDNA				Microsatellites					
	*n*	*np*	*h* ± SD	π± SD	*A* ± SD	*Ap*	*H_O_* ± SD	*H_E_* ± SD	*F_IS_*	*Welch p-value*
BP	5	2	0.600±0.101	0.0018±0.0006	7.583±3.298	2	0.808±0.163	0.756±0.095	0.1170**	n.p.
VC	10	6	0.800±0.089	0.0031±0.0005	6.333±2.498	1	0.633±0.164	0.715±0.105	–0.0699	n.p.
SJ	3	0	0.800±0.164	0.0019±0.0005	4.500±1.624	2	0.677±0.268	0.686±0.110	0.0809*	n.p.
ML	7	2	0.810±0.053	0.0031±0.0005	7.167±2.918	5	0.699±0.141	0.741±0.115	0.0579*	0.294
TP	5	2	0.805±0.050	0.0036±0.0006	6.750±3.279	5	0.624±0.187	0.706±0.121	0.1177**	0.945
PK	6	1	0.726±0.090	0.0022±0.0003	6.750±2.768	3	0.694±0.130	0.715±0.109	0.0252	0.734
TF	4	2	0.363±0.131	0.0007±0.0003	4.417±1.975	1	0.570±0.200	0.567±0.180	–0.0054	n.p.
SI	3	0	0.615±0.063	0.0034±0.0002	6.167±2.443	2	0.660±0.159	0.715±0.095	0.0791**	

The right most column shows the result of Welsh’s t-test between the populations of Staats Island and each its three potential continental source populations. *n*: number of haplotypes; *np*: number of private haplotypes; *h*: haplotype diversity*; π*: nucleotide diversity; *A*: mean number of alleles per locus; *Ap*: privates alleles; *He*: mean expected heterozygosity; *Ho*: mean observed heterozygosity; *Welch p-value*: significance value of Welch's t-test between the SI population and each of the other populations (e.g. Welch t-test between SI and ML has a *p-value* of 0.2948), *n.p.*: test not performed. Deviations from zero *p<0.05, **p<0.001.

### Microsatellite diversity

Among the 166 replicated microsatellite genotypes, we found two pairs of samples with the same allelic profiles suggesting that two individuals had been sampled twice. For each of these individuals one of the samples was discarded. Combining the continental and Staats Island samples, 150 alleles were detected in the 12 polymorphic loci genotyped from 164 guanacos. The number of alleles per locus ranged from 3 to 23, and the average number of private alleles per population was 2.6. Within populations, we found no significant departures from HWE equilibrium (*P*>0.0011 after Bonferroni correction), and no significant evidence of linkage disequilibrium among loci (*P*>0.0005 after Bonferroni correction for each pair of loci across all populations). Consistent with the other measures of genetic variability, we found high levels of population expected heterozygosity (0.70±0.058**,** mean and standard deviation respectively) and high values of mean number of alleles per locus (6.2±1.17) (Table 3).

While there was variation in the expected heterozygosity per population, these differences were not significant between the continental sample set considered as a whole and the Staats Island population (Welch t-test p-value  = 0.5424), or between the latter population and any of the continental populations (i.e. Monte Leon, Torres del Paine and Pali-Ayke) likely to be the source for the Staats Island population (Table 3). While other pairwise comparisons revealed no significant differences in *H_e_* between populations, it is still interesting that, consistent with the mtDNA results, the Staats Island population seems to present a higher *H_e_* and average number of alleles per locus than the Tierra del Fuego population (Table 3). However, a global test indicated a significant heterozygote deficiency in Bosques Petrificados, San Julian, Monte León, Torres del Paile and Staats Island as indicated by a positive *F_IS_* (Table 3).

### Genetic structure and population differentiation

The population comparisons show, with both types of markers, low levels of genetic differentiation among the continental samples (average *φ_ST_* = 0.026±0.069 and average *F_ST_* = 0.06±0.029; Table 4). Interestingly, the average divergence between the Staats Island population and the continental populations was not significantly different from the average divergence between the continental populations when measured with the microsatellites (Welch t-test p-value: 0.82). However, the same test based on the estimates from the mtDNA data showed a significantly higher divergence between Staats Island and the continental populations than between the continental populations (Welch t-test p-value: 0.0002). In contrast, the Tierra del Fuego population presented a significantly higher divergence with respect to the continent than did Staats Island for the microsatellites (Welch t-test p-value: 0.0002), while for the mtDNA data it was as divergent from the continental populations as the Staats Island population (Welch t-test p-value: 0.413). Consistent with the stronger effect of drift on island populations, the divergence between the Tierra del Fuego population and Staats Island was among the highest values of divergence observed with each marker type (Table 4).

**Table 4:** Pairwise population differentiations between guanaco populations.

Localities	BP	VC	SJ	ML	TP	PA	TF	SI
BP	-	0.0567*	–0.0424	0.0369	0.0702	–0.0219	0.4185**	0.3747**
VC	0.0542**	-	0.0842	0.1183**	0.1522**	0.0712*	0.4623**	0.3775**
SJ	0.1116**	0.0734**	-	–0.0679	–0.0629	–0.0590	0.3215**	0.2284*
ML	0.0678**	0.0490**	0.1035**	-	0.0225	–0.0166	0.1935**	0.1942**
TP	0.0464**	0.0422**	0.0949**	0.0297**	-	0.0536	0.2421**	0.1953**
PA	0.0489**	0.0425**	0.1007**	0.0291**	0.0213*	-	0.3190**	0.3112**
TF	0.1857**	0.1430**	0.1865**	0.1363**	0.1418**	0.1499**	-	0.3521**
SI	0.0835**	0.0697**	0.1138**	0.0334**	0.0408**	0.0463**	0.0937**	-

Genetic structure and statistical significance corrected by Bonferroni among Patagonia guanacos using mtDNA (pairwise *Ф_ST_* over diagonal) and microsatellite (*F_ST_* below diagonal) markers. Deviations from zero *p<0.05, **p<0.001.

**Figure 3:**
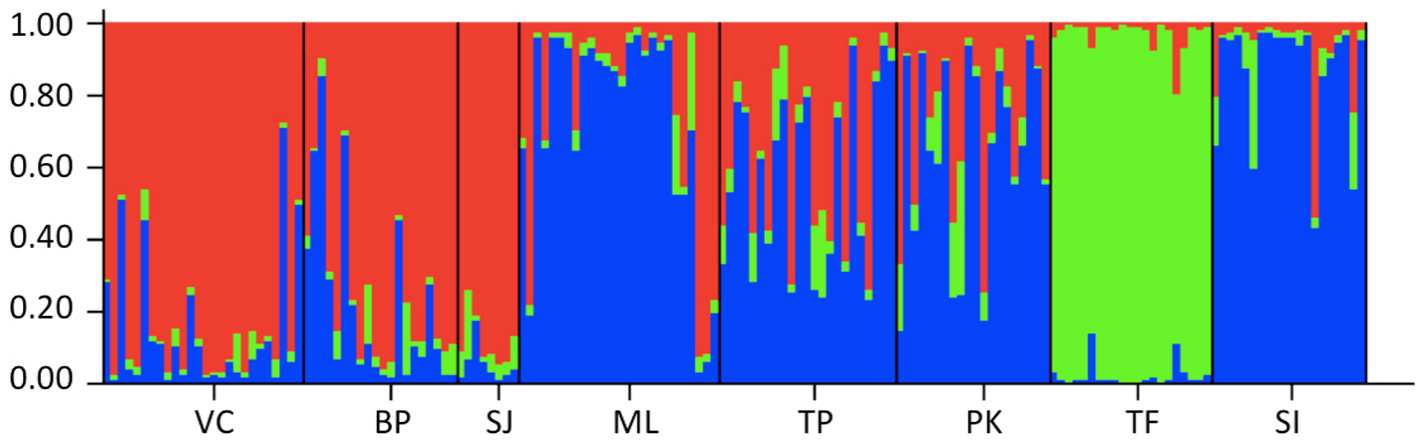
Clustering solution of the Patagonia guanaco populations. Plot of posterior probability of assignment for 164 guanacos (vertical lines) to three genetic clusters based on Bayesian analysis of variation at 12 microsatellite loci. Individuals are grouped by locality, and localities are indicated along the horizontal axis. Genetic Cluster 1: North Patagonia group (VC, BP, SJ); Genetic Cluster 2: Tierra del Fuego group (TF); Genetic Cluster 3: South Patagonia group (ML, TP, PK, SI). Population name abbreviations follow Table 1.

The STRUCTURE analysis suggested that a partition of the microsatellite dataset into three clusters (K = 3) had the highest posterior probability, as measured with the ΔK Evanno method. The three clusters corresponded to the group of samples from i) northern Patagonia, ii) the samples from southern Patagonia including the guanacos from Staats Island, and iii) the samples from the island of Tierra del Fuego (Figure 3).

### Genetic Origin of the Staats Island population

Haplotypic diversity indicated that Monte Leon is the most similar continental population to Staats Island. The island population had three haplotypes, which were observed together only in the Monte Leon population. In comparison to Monte Leon, no other population presented more than one haplotype shared with the Staats Island. Moreover, the dominant haplotype in Staats Island (frequency of Hap_20 = 0.54) was only recorded in the Monte Leon population, where it occurred at a moderately low frequency (0.09). This finding was supported by assignments tests, which showed that most of the Staats Island individuals could be assigned to the Monte Leon population.

Of the 164 individuals analysed in the STRUCTURE analyses, 125 were assigned to a single cluster with a q value greater than 75%. Twenty-two percent of these individuals were assigned to the Tierra del Fuego cluster, while 39% were assigned to either the North Patagonia cluster or the South Patagonia cluster. Out of the 20 Staats Island individuals, 19 were assigned to the South Patagonian cluster. However, of these samples only 16 presented a *q* value higher than 75% (Table 5). The only Staats Island individual not assigned to South Patagonia was assigned by STRUCTURE to North Patagonia but with a q value of 0.56. The BAPS and GENECLASS analyses placed all the Staats Island individuals in the South Patagonia cluster. However, while both methods assigned most of the Staats Island individuals to the population of Monte Leon (65%), BAPS and GENECLASS assigned between 5 and 7 individuals to the other two South Patagonia clusters/populations (Table 5).

**Table 5:** Results of population assignment algorithms STRUCTURE, BAPS and GENECLASS for guanacos of Staats Island.

Staats Island Individuals	STRUCTURE		BAPS		GENECLASS	
	Population assignment	*q*	Population assignment	Posterior probability	Population assignment	Likelihood Ratio
1	South Patagonia	0.665	TP	1.0	TP	12,384
2	South Patagonia	0.968	ML	1.0	ML	16,604
3	South Patagonia	0.965	ML	1.0	ML	15,898
4	South Patagonia	0.976	ML	1.0	ML	14,999
5	South Patagonia	0.895	ML	1.0	TP; ML	15,911; 15,977
6	South Patagonia	0.697	TP	1.0	TP; ML	11,614; 11,847
7	South Patagonia	0.981	ML	1.0	PK; ML	18,382; 18,751
8	South Patagonia	0.980	ML	1.0	ML	17,130
9	South Patagonia	0.973	ML	1.0	ML; PK	15,190; 15,269
10	South Patagonia	0.973	ML	1.0	TP	14,056
11	South Patagonia	0.972	ML	1.0	ML	16,778
12	South Patagonia	0.959	ML	1.0	PK	17,717
13	South Patagonia	0.979	ML	1.0	PK; ML	18,475; 18,710
14	North Patagonia	0.558	ML	1.0	ML	18,140
15	South Patagonia	0.896	PK	1.0	PK	13,829
16	South Patagonia	0.915	TP	1.0	TP	14,000
17	South Patagonia	0.966	ML	1.0	ML	17,048
18	South Patagonia	0.974	ML	1.0	ML	16,695
19	South Patagonia	0.577	TP	1.0	TP	11,602
20	South Patagonia	0.962	ML	1.0	ML	16,328

### Bottleneck and *N_e_* of Staats Island population

The Staats Island population presented evidence of having passed through a bottleneck under the TPM model (Table 6). In contrast, none of the continental population showed evidence of having passed through a bottleneck. While this result may indicate some evidence of a deviation from mutation drift equilibrium in the Staats Island population, it is unexpected that after a bottleneck the genetic diversity (e.g. number of alleles per locus) in this isolated island population would remain within the range of that in the continental populations (Table 3).

**Table 6:** Summary of parameters and results for the *M*-ratio and BOTTLENECK analyses used to detect significant reductions in effective population size.

Localities					Bottleneck	
	*N_e_*	θ	*M*-ratio	*M_c_*	Mutation model	Heterozygote excess
BP	5	0.01	0.8248	0.8666	TPM	*P* = 0.1696
	50	0.1	0.8248	0.8527	SMM	*P* = 0.8493
	500	1	0.8248	**0.7826**		
	1,556*	3.11	0.8248	**0.7184**		
	5,000	10	0.8248	**0.6572**		
VC	5	0.01	0.6539	0.8626	TPM	*P* = 0.1901
	50	0.1	0.6539	0.8541	SMM	*P* = 0.5151
	500	1	0.6539	0.7871		
	1,093*	2.18	0.6539	0.7450		
	5,000	10	0.6539	0.6779		
SJ	5	0.01	0.7227	0.8626	TPM	*P* = 0.1018
	50	0.1	0.7227	0.8527	SMM	*P* = 0.6889
	500	1	0.7227	0.7741		
	1,261*	2.52	0.7227	**0.7067**		
	5,000	10	0.7227	**0.5772**		
ML	5	0.01	0.8015	0.8626	TPM	*P* = 0.51514
	50	0.1	0.8015	0.8527	SMM	*P* = 0.96802
	500	1	0.8015	**0.7837**		
	1,202*	2.4	0.8015	**0.7374**		
	5,000	10	0.8015	**0.6683**		
TP	5	0.01	0.7964	0.8666	TPM	*P* = 0.2119
	50	0.1	0.7964	0.8540	SMM	*P* = 0.6889
	500	1	0.7964	0.7873		
	1,522*	3.04	0.7964	**0.7249**		
	5,000	10	0.7964	**0.6691**		
PK	5	0.01	0.7088	0.8611	TPM	*P* = 0.3667
	50	0.1	0.7088	0.8527	SMM	*P* = 0.9959
	500	1	0.7088	0.7782		
	1,276*	2.55	0.7088	0.7299		
	5,000	10	0.7088	**0.6549**		
TF	5	0.01	0.6732	0.8657	TPM	*P* = 0.3667
	50	0.1	0.6732	0.8531	SMM	*P* = 0.9959
	500	1	0.6732	0.7865		
	661*	1.32	0.6732	0.7690		
	5,000	10	0.6732	**0.6598**		
SI	5	0.01	0.7565	0.8626	TPM	***P*** = **0.0067**
	50	0.1	0.7565	0.8527	SMM	*P* = 0.1330
	500	1	0.7565	0.7833		
	661*	1.32	0.6732	0.7690		
	5,000	10	0.7565	**0.6589**		

Bold indicates the *M*-ratios used the *N_e_* from *N_e_* = *H_E_*/4μ (1 – *H_E_*) indicated by *, and the bottleneck signature in bold indicates significance (*P*<0.05). *M_c_* = *p_s_* = 0.9 and Δ_g_ = 3.5.

The BOTTLENECK analysis was complemented with the M-ratio method [64]. For this method we estimated a pre-bottleneck *N_e_*, which, based on the expected heterozygosity of the Staats Island population, resulted in values ranging between 661 to 1556 individuals [65]. While these values may seem large at first glance, they are not extremely different than our own field observations, which show that up to almost 400 individuals existed in the island by December 2003 [71]. Thus, our biologically plausible pre-bottleneck Ne values for the M-ratio analysis were set to 5, 50, 500, and 5,000 (Table 6). The population size of five represents the most liberal test for significant reductions in population size by effectively reducing the pre-bottleneck to *θ*, thereby increasing *M_c_* values. However, we also used *N_e_* values considerably larger than our estimates of the current *N_e_*. Among the various continental populations only the Valle Chacabuco (Chile) population showed no evidence of bottleneck for any of the *N_e_* values tested. In contrast, all other populations show M-ratio values below *M_c_* indicating they may have passed through a bottleneck. However, these analyses seem to become only significant for large values of simulated *N_e_*. Although the observed *N_e_* estimates of our populations based on the expected heterozygosity sometimes fell within the range of the simulated *N_e_* values that were significant, it is unlikely that such a large *N_e_* currently characterizes our populations. This is particularly true for the Staats Island population, since the demographic records since its establishment have never exceeded more than 400 animals.

### Effect of Population variation on genetic diversity detected by simulation

**Figure 4:**
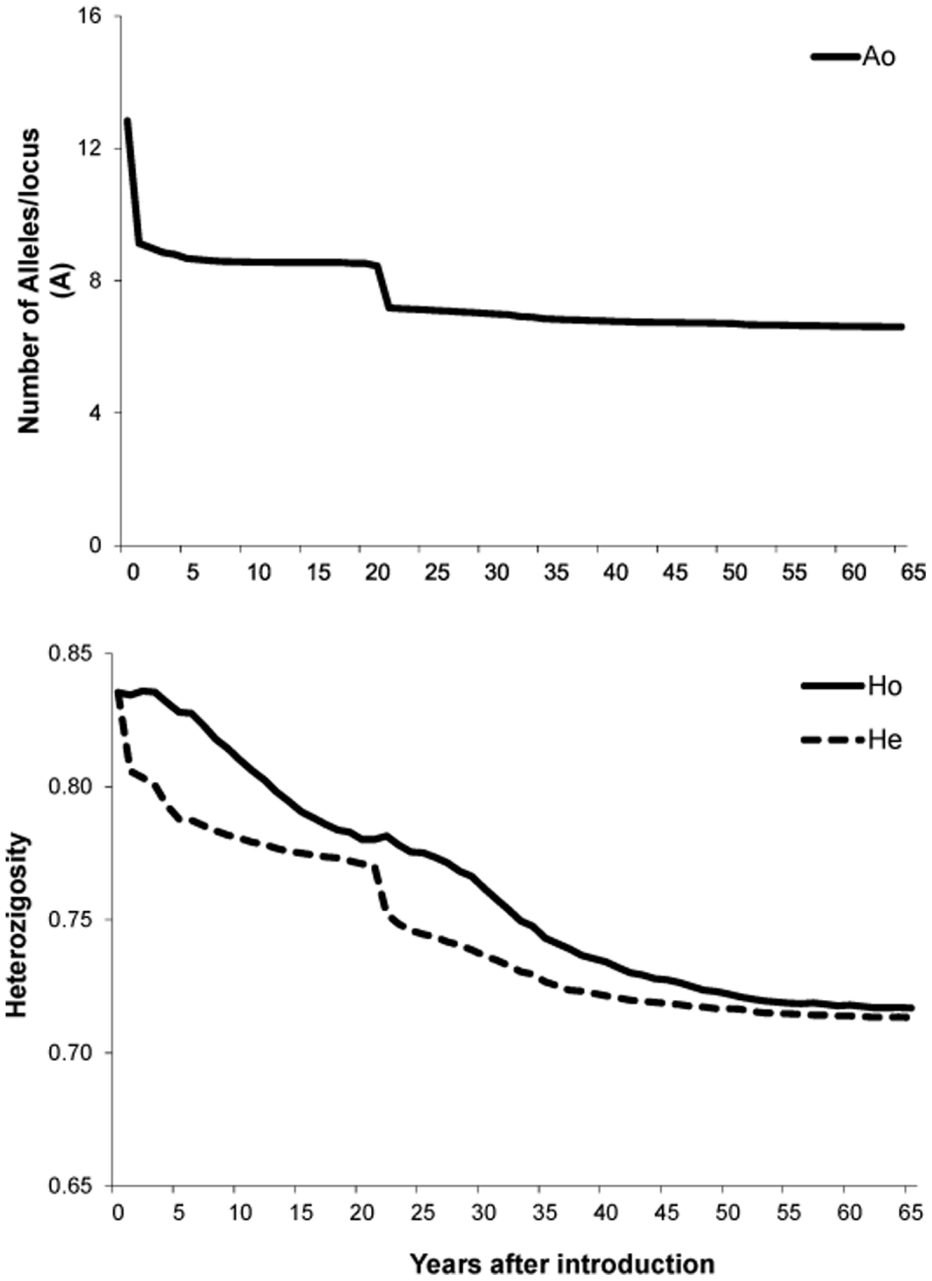
Temporal changes in observed alleles per locus (solid line, *A_O_*), and observed (solid line, *H_O_*) and expected (spotted line, *H_E_*) heterozygosity as predicted by a simulation of the guanaco population after introduction in Staats Island. In the simulation, year 0 represents the pre-bottleneck (source in mainland) population and the bottleneck occurred in years 1 and 22. Population size changed as indicated in Figure 2.

A population of 10,000 individuals was simulated on the basis of the allele frequencies detected in Patagonia. This population was bottlenecked to 15 animals (assuming 8 females) in order to mimic the founding of the Staats Island population 65 years ago (Figure 2). As expected, after the bottleneck a drastic reduction by ∼29% in the mean number of alleles was observed from 12.8 (SE = 0.29) to 9.1 (0.16) (Figure 4), while the *H_E_* decreased by only 3.5% from 0.84 (SE = 0.01) to 0.81 (SE = 0.01) (Figure 4). A second bottleneck after ∼25 years since the introduction of guanacos to the island further reduced the allelic diversity to approximately 51% of the original variation (i.e. 6.6 (SE = 0.61)), although it had no effect on *H_E_*. After the initial bottleneck *H_E_* steadily decreased throughout the simulation until reaching a value of 0.71 (SE = 0.01) at the end of the simulations.

## Discussion

### Genetic diversity and population structure

The guanacos of the Staats Island were introduced to Falkland/Malvinas archipelago ∼75 years ago, with our samples representing animals ∼65 years after the original introduction. This population was started from a founding group of animals no larger than 15 animals [38], [40]. While the initial guanaco population prospered on the island, the number of animals was dramatically reduced ∼25 years later as part of a plan to eradicate the population. This history of severe demographic reductions accompanied by isolation from other guanaco populations, suggests the Staats Island population should present a significant reduction in genetic variation due to the strong effect of genetic drift. Nevertheless, the analysis of mitochondrial and nuclear DNA variation in the southern South American guanaco populations that were examined showed unexpected and similar patterns of genetic variation and lack of divergence between the island and continental populations.

The Staats Island guanaco population is currently at similar levels of genetic diversity compared to continental natural populations in Patagonia and is higher than those measured in the Tierra del Fuego population despite its large size of ∼80,000 animals. Although there are various mechanisms that could explain the retention of high genetic diversity in Staats Island (e.g. natural selection favouring diversity), the most likely candidate is the high genetic diversity due to a random sampling of individuals from the continental population, which were already diverse (Table 3, [36]).

The guanaco of southern South America were subdivided into three genetic populations that present various levels of divergence. The Staats Island was assigned to the clusters of South Patagonia, which presents the potential source populations from which its founder individuals were suspected have been taken [38]. Interestingly, the Tierra del Fuego island population appears to be a separate cluster that shows little admixture with continental populations. Although this may initially seem surprising, it is expected since this population was founded approximately 10,000–11,000 years ago and isolated 8,000 years ago when land bridges connected this island and the continent [25]. Consequently, this population has become genetically isolated from the continent [36] as no restocking of lost genetic variation has occurred via gene flow, and genetic drift has changed the population distribution of allelic/haplotypic variants. In contrast, the guanacos of Staats Island still remain genetically similar to the continental populations because of its short evolutionary history (∼65 years).

### Demographic history of the Staats Island population

The guanaco population on Staats Island surprisingly presented similar patterns of divergence as were observed in the various continental populations. Its nuclear DNA showed an average divergence from the continental populations similar to that between continental populations. This observation likely reflects the large genetic variation of Staats Island’s founding population and a reduced effect of genetic drift on the distribution of allelic frequencies over the past ∼65 years, as observed in other species [72].

Nevertheless, this finding was unexpected due to the recorded demographic history of Staats Island’s guanaco population, which was founded by few animals [15] and later dramatically reduced in the early 1960s to approximately 10 – 20 individuals [38], [39]. However, our analyses failed at detecting a consistent bottleneck signature in the Staats Island population. It is likely that if the demographic change had little effect on its genetic diversity (i.e. the *N_e_* reduction was not very large and/or the reduction was not sufficiently long), the signature of a distant bottleneck cannot be picked up anymore [73]. Additionally, if after the demographic reduction the population grew exponentially, any weak signature left by the bottleneck was likely erased from the population’s genetic make-up (Figure 2). As the bottlenecks experienced by the Staats Island guanaco population were only a few generations long and in both cases were followed by exponential demographic increases, it is expected that the bottleneck signatures were at least partially erased [74]. Although it is strongly suspected that young guanacos less than one year old were originally introduced to Staats Island [38], if older females were introduced and pregnant, then the effective number of individuals brought to the island would have been larger.

As revealed by the nuclear DNA analysis, genetic drift appears to have not played a major role in the microevolution of the guanaco population on Staats Island, yet it certainly had a role as observed in the mtDNA analyses. The mtDNA typically has a fourfold smaller *N_e_* than the nuclear DNA in diploid species [75] and genetic drift is expected to have a larger effect on mtDNA. Consistent with this expectation, we found a significantly higher average divergence between Staats Island and the continental populations with the mtDNA compared with the microsatellite size variation (Welch t-test p-value: 0.00097). However, this estimate of divergence did not significantly differ between the continental populations and the island population of Tierra del Fuego. Both islands present an average divergence from continental populations approximately 12 times higher than the continental populations show with respect to each other (*F_ST_* = 0.303 and 0.026 respectively). Moreover, it is expected that the two island populations, with their smaller *N_e_* (relative to the continent), have drifted from each other even more than they did from the continental population, and as expected, they presented the highest pairwise population differentiation (*φ_ST_* = 0.53, Table 4).

### Origin of the introduced guanaco population

A continental-coastal origin of current guanacos in Staats Island is supported by shared haplotypes and results of assignment tests using microsatellites markers. The animals that were brought to Staats Island were shipped from the Rio Gallegos port in Argentina in two separate years, i.e. 5 animals in 1938 and 10 animals in 1939, but it is not clear from where they were collected [38]. It is known that at the time a guanaco population existed near Puerto Gallegos, but since then it has become locally extirpated or geographically displaced (WF pers. obs.). Today, the closest extant guanaco population to Rio Gallegos is Monte León, approximately ∼150 km north. Consistent with an origin from South Patagonian populations, the Staats Island population groups within the cluster of South Patagonian populations (Monte León, Torres del Paine, and Pali-Ayke). Moreover, the Staats Island population shares all of their haplotypes with Monte León (MT), including its most frequent haplotype (H 20), which only occurs in Southern South America at Monte León. Nevertheless, Torres del Pine and Pali-Ayke also share one of its haplotypes with Staats Island, and as shown by the assignment tests, at least some animals can be placed in these two populations with high probability (Table 5). Thus, we conclude that while most of the genetic background of Staats Island appears to have originated in Monte León (or a Monte León-like population; Table 2 and 5), it is possible that some of the extant genetic variation originated from neighbouring South Patagonian populations, confirming historical records [38].

## Conclusions and Conservation Implications

The Staats Island guanaco population presents a similar amount of genetic variation as continental guanaco populations. Interestingly and unexpectedly, this guanaco island population has been resilient to the effect of dramatic demographic changes as its genetic variation is not yet depauperated, i.e. become impoverished. Nevertheless, this by no means implies that potential severe cullings or hunts would not diminish the population’s fitness. It is likely that the two dramatic population size reductions did not significantly affect the Staats Island population because the highly reduced numbers lasted only a few generations and surviving animals were a sufficiently large random sample of the pre-bottleneck genetic variation.

Similarly, continental guanaco populations also harbor high levels of genetic variation despite of the extensive pressure that was put on these populations in the recent past. Continental guanacos were hunted to increase the area intended for sheep flocks, because of a perceived forage competition between guanacos and sheep, and for the economic value of their pelts [17], [76], [77]. Chile exported some 35,000 pelts during the 20th century [78] and Argentina 223,000 units in only four years from 1976 to 1979 [77]. The vast majority of these pelts were obtained in Patagonia. Therefore, the current guanaco populations in Patagonia are survivors of massive hunting mainly on chulengos in the recent past, and the subdivision between the North and South Patagonia cluster probably reflects separate groups of animals that persisted throughout the last century, resulting in reconstituted extant populations. Currently, the biological and socio-economic worth of the Staats Island guanacos is high. The silk-like wool of the guanaco is worth US$60 to 80 per kg, an economic reality that has prompted Argentina’s current management of Patagonian guanaco that is harvesting wool from annual, live-capture roundups [79]. The future of the privately owned guanacos on Staats Island is unknown, but its intrinsic and economic value is unmistakable.

Compared with the mainland, the relative population decrease on Staats due to killing is substantially higher when it was reduced to 15–20 animals. Therefore, it is surprising that this guanaco population has maintained genetic diversity much more than expected according its demographic history of two dramatic reductions in size. This has revealing implications and hope for surviving small remnant populations on the mainland that could be maintaining genetic diversity in spite of their low numbers. Nevertheless, it is important that although Staats Island and Patagonia guanacos harbor relatively high genetic variation, that their population numbers not be allowed to decline to severely low levels to avoid unforeseen, adverse-stochastic ecological events. Currently, guanacos in Staats Island are suffering neonatal malformations and mortality [38].

A genetic assessment, ideally with genomic-level data, of critically small and isolated guanaco populations from throughout their natural distribution [20], as well as populations established from translocation programmes of few individuals or frequency of reintroductions [31], [32], is needed to document existing population sizes and biogeographic patterns. This would assist in ensuring a correct interpretation of guanaco genetic patterns and space in establishing and assessing informed and coordinated multidisciplinary management plans for the long-term recovery of guanaco populations.
